# Genetic Diversity and Population Structure of Ethiopian Sheep Populations Revealed by High-Density SNP Markers

**DOI:** 10.3389/fgene.2017.00218

**Published:** 2017-12-22

**Authors:** Zewdu Edea, Tadelle Dessie, Hailu Dadi, Kyoung-Tag Do, Kwan-Suk Kim

**Affiliations:** ^1^Department of Animal Science, Chungbuk National University, Cheongju, South Korea; ^2^International Livestock Research Institute, Addis Ababa, Ethiopia; ^3^Ethiopian Biotechnology Institute, Addis Ababa, Ethiopia; ^4^Department of Animal Biotechnology, Faculty of Biotechnology, Jeju National University, Jeju, South Korea

**Keywords:** Ethiopian sheep, high-density chip, genetic diversity, population structure, SNP

## Abstract

Sheep in Ethiopia are adapted to a wide range of environments, including extreme habitats. Elucidating their genetic diversity is critical for improving breeding strategies and mapping quantitative trait loci associated with productivity. To this end, the present study investigated the genetic diversity and population structure of five Ethiopian sheep populations exhibiting distinct phenotypes and sampled from distinct production environments, including arid lowlands and highlands. To investigate the genetic relationships in greater detail and infer population structure of Ethiopian sheep breeds at the continental and global levels, we analyzed genotypic data of selected sheep breeds from the Ovine SNP50K HapMap dataset. All Ethiopian sheep samples were genotyped with Ovine Infinium HD SNP BeadChip (600K). Mean genetic diversity ranged from 0.29 in Arsi-Bale to 0.32 in Menz sheep, while estimates of genetic differentiation among populations ranged from 0.02 to 0.07, indicating low to moderate differentiation. An analysis of molecular variance revealed that 94.62 and 5.38% of the genetic variation was attributable to differences within and among populations, respectively. Our population structure analysis revealed clustering of five Ethiopian sheep populations according to tail phenotype and geographic origin—i.e., short fat-tailed (very cool high-altitude), long fat-tailed (mid to high-altitude), and fat-rumped (arid low-altitude), with clear evidence of admixture between long fat-tailed populations. North African sheep breeds showed higher levels of within-breed diversity, but were less differentiated than breeds from Eastern and Southern Africa. When African breeds were grouped according to geographic origin (North, South, and East), statistically significant differences were detected among groups (regions). A comparison of population structure between Ethiopian and global sheep breeds showed that fat-tailed breeds from Eastern and Southern Africa clustered together, suggesting that these breeds were introduced to the African continent via the Horn and migrated further south.

## Introduction

In the Horn of Africa and especially in Ethiopia where the economy is predominantly agriculture-based, sheep and their products play a critical role in the livelihood of millions of farmers and pastoralists (Wilson, 2011). Sheep serve as a source of income, mutton, and manure; provides an economic buffer in the event of crop failures; and fulfill many other socio-cultural functions. In some areas such as the cool alpine and arid lowlands where crop production is not a viable economic option, sheep production is the sole option for livelihood (Tibbo, 2006). Sheep are also important for the national economy; indigenous populations have evolved in diverse and harsh environments in Ethiopia where they face disease and parasite burdens, feed shortage, and extreme temperatures. Consequently, these animals likely harbor gene variants uniquely adapted to specific environmental conditions that may not be present in commercial breeds. The economic and agricultural value of sheep is expected to increase as a result of climate change (Seo, 2008; Seo et al., 2010); genetic characterization of local breeds adapted to extreme environments using modern genomic tools can ensure the breeding of hardy sheep populations (Boettcher et al., 2015; Yang et al., 2016).

Given its proximity to the Arabian Peninsula, Ethiopia is considered as a genetic corridor for the introduction of livestock species including sheep to the African continent (Hanotte et al., 2002; Muigai and Hanotte, 2013). Extensive hybridization has occurred between sheep breeds introduced at various times via different routes, making the Horn Africa in general and Ethiopia in particular an excellent resource for the study of genetic diversity in domestic livestock breeds. The ecological, climatological, ethnic, and cultural diversity of Ethiopia is reflected in its large sheep populations (25.5 million heads) (Leta and Mesele, 2014), which can be phenotypically classified into 14 native populations (Gizaw et al., 2007) in addition to populations distributed along the northern, southwestern, and western borders of the country that have yet to be described. These local populations are mainly named after the geographic location or ethnic group/community rearing them, or based on phenotypic characteristics; for instance, the 14 Ethiopian sheep populations are broadly categorized according to their tail phenotypes as thin- tailed (one breed), fat-tailed (11 populations), and fat-rumped (two populations) (Gizaw et al., 2007). The short fat-tailed population mainly inhabits the sub-alpine regions; long fat-tailed sheep are predominant in mid- to high-altitude environments; and fat-rumped sheep are distributed in dry lowland areas (Gizaw et al., 2007).

Characterizing genetic diversity is a key aspect of developing sustainable breed improvement strategies (Groeneveld et al., 2010) and understanding adaptation to extreme environments (Boettcher et al., 2015). Although several studies have investigated the origin of African sheep breeds, many breeds and populations have yet to be fully characterized. The genetic diversity and population structure of Ethiopian sheep populations have been examined using non-recombinant (mitochondrial DNA) and selection-neutral markers (Gizaw et al., 2007; Helen, 2015). However, microsatellite-based studies have provided limited global picture as it included only local sheep breeds of Ethiopia. In general, at the African continent level, there have been far fewer studies on sheep diversity and population structure using genome-wide nuclear markers as compared to non-recombinant markers (Muigai, 2003a; Bruford and Townsend, 2006; Aswani, 2007; Horsburgh and Rhines, 2010; Helen, 2015). It was recently reported that genetic diversity estimated using microsatellites was not correlated with genome-wide single nucleotide polymorphism (SNP) diversity estimates, with larger genetic differentiation values obtained by the former approach (Ciani et al., 2013; Fischer et al., 2017). On the other hand, the large number of genome-wide SNP markers makes it superior to microsatellites for inferring population structure (Glover et al., 2010; Gärke et al., 2012). The recently developed genome-wide high-density ovine SNP array has provided a tool for investigating genetic diversity at a high resolution, inferring population history, and mapping genomic regions subject to selection and adaptation (Kijas et al., 2009; Yang et al., 2016; Zhao et al., 2017). Despite the richness of Ethiopia’s sheep genetic resources, only one population was represented in previous genome-wide global sheep analyses (Kijas et al., 2009). Therefore, the extent of genetic variation and patterns of admixture are not known for most Ethiopian sheep populations. Additionally, polymorphisms in the Ovine HD chip in non-reference African/Ethiopia sheep populations have not been identified or validated.

The present study provides the first analysis of high density (∼600K) ovine SNPs in Ethiopian sheep breeds. We sampled and genotyped five Ethiopian sheep populations adapted to diverse agro-ecologies using the Infinium HD SNP BeadChip (600K). A detailed understanding of the genetic landscape of national populations requires sampling of representative breeds from wider geographic regions, particularly from a center of domestication and along migration routes (Zhao et al., 2017). To establish historical patterns of admixture and the genetic relatedness of Ethiopian sheep breeds on a broader geographic scale, we compared these breeds with 12 others extracted from Ovine SNP50K HapMap datasets as well as one from Morocco, the data for which was generated by the NextGen Consortium. Two North African sheep breeds (Egyptian Barki and Moroccan) were not previously analyzed (Kijas et al., 2012) but were included here to examine their genetic influence on Ethiopian/East African sheep genetic composition.

## Materials and Methods

### Breeds/Populations and Samples

Nasal samples were collected using Performagene LIVESTOCK’s nasal swab DNA collection kit (DNA Genotek, Kanata, ON, Canada) from a total of 72 animals representing five Ethiopian sheep populations: Arsi-Bale, Horro, Menz, Adilo, and Blackhead Somali. Three of these (Horro, Arsi-Bale, and Adilo) are long fat-tailed hairy sheep; Menz is a short fat-tailed coarse-wool sheep; and Blackhead Somali belong to the fat-rumped group (Gizaw et al., 2008). Both female and male animals were randomly sampled from multiple flocks.

Blackhead Somali sheep (also known as Blackhead Ogaden or Berbera Blackhead) exist at low altitudes (500–1000 m above sea level [a.s.l.]) and are well adapted to arid and semi-arid environments characterized by high ambient temperature, low precipitation (200–400 mm), and recurrent drought (Wilson, 1991). The breed is distinguished by the absence of horns in both sexes, black head and neck and white body and limbs, and a fat rump (Wilson, 2011), and is reared across the Horn of African (Ethiopia, Djibouti, Somali, Kenya, and Sudan) under a mobile pastoral management system that includes heavy heat stress, long walks in search of pasture and water, long watering intervals, and few health management practices. In contrast, three of the populations (Horro, Arsi-Bale, and Menz) are reared under sedentary farming systems. Horro sheep are mainly distributed throughout western and southwestern parts of the country inhabiting mid- to high-altitude (1400–2000 m a.s.l.) areas with a mean precipitation of 1000–2000 mm. Horro sheep are characterized by a larger body size and higher twinning rate than other indigenous breeds (Gizaw et al., 2013). Arsi-Bale is the predominant breed in the eastern and south-central parts of Ethiopia, spreading from the Central Great Rift Valley to the Bale mountains (>3000 m a.s.l.). Menz sheep have a relatively small body size with an average live weight of 20.1 ± 0.3 kg, are raised for meat and coarse wool production, and are well adapted to cool highland areas (2500–3000 m a.s.l.) (Haile et al., 2002; Gizaw et al., 2008; Getachew et al., 2015). The Adilo (Wolaita) sheep breed is distributed in southern Ethiopia and characterized by long fat-tail and large body size (Melesse et al., 2013). Phenotypic descriptions and environmental variables of the study sheep populations are summarized in **Table 1**.

**Table 1:** Summary of phenotypic characteristics and environmental variables of the study sheep populations.

Phenotypes and environmental variables	Population				
	Adilo (Wolaita)	Arsi-Bale	Horro	Blackhead Somali	Menz
Tail-type	Long fat-tail	Long fat-tail	Long fat-tail	Short fat-rump	Short fat-tail
Body weight (kg)	27.40–28.10	26.96–28.60	30.71–35.40	24.92–27.90	20.1
Coat color	Brown, white, gray, and combinations of different colors	Brown, red, black, gray, white, and combinations of different colors	Brown, creamy white/fawn	White body, black head and neck	Black, brown, white and combinations of different colors
Horn	Male short horned, most females polled	Males and most females are horned	Polled	Polled	Horned
Altitude (m)	1618–2043	2492–2810	1400–2000	500–1500	2500–3000
Rainfall (mm)	1175–1449	1020–1076	1000–2000	200–400	900–1102
Temperature (°C)	13.60–25.60	7.87–22.20	10.62–22.53	14.40–35.0	7.6–22.1
Agro-ecology	Wet highland	Wet highland	Wet highland	Arid-lowland	Sub-alpine
Use	Meat	Meat	Meat	Meat	Meat and wool
Management	Mixed crop-livestock	Mixed crop-livestock	Mixed crop-livestock	Pastoral/agro-pastoral	Sheep-barley
Community	Wolaita/Hadiya	Oromo	Oromo	Oromo/Somali	Amhara

*Source: Gizaw et al. (2008), Ethiopia Sheep and Goat Productivity Improvement Program [ESGPIP] (2009), Melesse et al. (2013).*

To compare the genetic relationship between sheep breeds in Ethiopia and those on other continents and investigate historical patterns of admixture, we also used genotype data of 228 animals representing 12 breeds from North Africa, Middle East, South Africa, Europe, and Asia from the Ovine HapMap project (International Sheep Genomics Consortium^1^). We also included Moroccan sheep data generated by the NextGen Consortium^2^. Details regarding sample sizes, breeds, and geographic origins are summarized in **Table 2**.

**Table 2:** Diversity indices in 18 sheep breeds estimated from different single nucleotide polymorphism (SNP) datasets.

	SNP dataset							
Breed/population	Country/origin	*n*	497,294				40,770	
			PI_HAT	HO	HE	*F*	HO	HE
Adilo	Ethiopia/Africa	11	0.09	0.30	0.30	–0.01	0.30	0.30
Arsi-Bale	Ethiopia/Africa	8	0.09	0.30	0.29	–0.03	0.30	0.29
Blackhead Somali	Ethiopia/Africa	15	0.094	0.32	0.30	–0.04	0.31	0.30
Horro	Ethiopia/Africa	15	0.03	0.3	0.31	0.02	0.30	0.31
Menz	Ethiopia/Africa	12	0.08	0.33	0.32	–0.04	0.33	0.31
Overall		61	0.01	0.31	0.33	0.06	0.31	0.33
Red Maasai	Africa/Kenya	20					0.33	0.32
Namaqua Afrikaner	South Africa	12					0.29	0.24
African Dorper	South Africa	21					0.34	0.33
Egyptian Barki	Egypt/Africa	12					0.35	0.35
Moroccan	Morocco/Africa	21					0.36	0.37
Local Awassi	Israel/Middle East	21					0.36	0.36
Afshari	Iran/Middle East	20					0.35	0.34
Indian Garole	Indian/Asia	20					0.29	0.29
Barbados Black Belly	Americas/Caribbean	20					0.32	0.33
Brazilian Creole	Brazil/Americas	20					0.33	0.36
Churra	SW Europe	20					0.36	0.36
Australian Merino	SW Europe	21					0.37	0.37
Dorset Horn	British/North Europe	21					0.32	0.29

*HE, expected heterozygosity; HO, observed heterozygosity; PI_HAT, average relatedness; SW, South-west.*

### Genotyping, Quality Control, and Markers Screening

Ethiopian sheep samples were genotyped with the Ovine Infinium HD BeadChip (Illumina, San Diego, CA, United States) by GeneSeek/Neogen (Lincoln, NE, United States). Among the 606,006 SNPs, 577,401 were autosomal, 1291 were unmapped to any ovine chromosome (OAR), and 27,314 were located on the X chromosome.

Autosomal SNPs with call rates <90% and minor allele frequency (MAF) <0.01 were filtered out, leaving 497,294 SNPs with average and median gaps of 4.92 and 3.58 kb, respectively. Additionally, 11 samples with call rates ≤ 85% were excluded from further analysis. To test for potential effects of ascertainment bias on diversity index estimates, 497,294 SNPs were subjected to linkage disequilibrium (LD) pruning using the parameter (50 5 0.20), yielding 80,602 SNPs.

Genotypic data for the 600K and 50K platforms were merged using SNP and Variation Suite v.8.5.0 (Golden Helix, Bozeman, MT, United States ^3^). A total of 41,752 SNPs overlapping between the two platforms were filtered according to quality control criteria; SNPs with call rates <90% and MAF <0.01 were removed, leaving 40,770 SNPs for subsequent analyses. A total of 6163 SNPs remained for population structure analysis after 40,770 SNPs in each population were pruned based on LD using the parameter (50 5 0.80).

### Statistical Analysis

#### Genetic Diversity

Minor allele frequency and deviation from Hardy–Weinberg equilibrium (HWE) were estimated by SNPs for each of the five Ethiopian sheep populations using SNP and Variation Suite v.8.5.0. Alleles were categorized into different bins based on their frequency: fixed alleles (MAF = 0.00), rare alleles (>0.00–<0.05), intermediate alleles (≥0.05–<0.10) and common alleles (≥0.10 and ≤0.5). Diversity indices were estimated from three datasets: (i) 497,294 SNPs that passed the quality control threshold of MAF ≥ 0.01 and call rate ≥ 90%; and (ii) 80,602 SNPs that remained after pruning 497,294 based on LD using the parameter (50 5 0.20) in SNP and Variation Suite v.8.5.0; and (iii) 40,770 SNPs common to 600K and 50K platforms.

To estimate within-population genetic diversity, we calculated observed heterozygosity (HO), expected heterozygosity (HE), and inbreeding coefficients for the three datasets using PLINK (Purcell et al., 2007). Animal relatedness was estimated as the proportion of gene identity-by-descent between sample pairs within the breed/population as an average relatedness (PI_HAT) value using the same software.

#### Genetic Relationships and Population Structure

Pairwise genetic differentiation (fixation index, *F*_ST_) (Weir and Cockerham, 1984) and Reynolds’ genetic distances (Reynolds et al., 1983) between all pairs of sheep populations were calculated using Arlequin v.3.5.2 (Excoffier and Lischer, 2010). The significance of genetic differences was determined from 10,000 permutation tests. Analysis of molecular variance (AMOVA) with 10,000 permutations was carried out using the same software. Using Reynolds’ genetic distance, a neighbor-net tree was constructed using SPLITTREE4 v.14.5 (Huson and Bryant, 2006).

Population genetic structure was assessed using STRUCTURE v.2.3.4 software (Pritchard et al., 2000) using a Bayesian model based on 80,602 SNPs in the five Ethiopian sheep populations and 18 breeds and on 6163 SNPs overlapping between OvineSNP50 and 600K and remaining after pruning based on LD. An admixture ancestry model with correlated allele frequencies was generated for a putative number of subpopulations (K) ranging from 2 to 18. Five runs of 20,000 Markov chain Monte Carlo iterations after a burn-in period of 10,000 iterations were carried out for each *K*-value. The STRUCTURE output was analyzed in HARVESTER (Earl, 2012). The most likely number of clusters was identified by the Δ*K* method (Evanno et al., 2005). Population structure was separately inferred by principal component analysis (PCA) based on 497,294 SNPs for the five Ethiopian sheep populations and 40,770 SNPs for all breeds using SNP and Variation Suite v.8.5.0.

## Results and Discussion

### Intra-population Genetic Variability

The mean MAFs for Arsi-Bale, Horro, Adilo, Menz, and Blackhead Somali sheep were 0.19 ± 0.16, 0.21 ± 0.16, 0.20 ± 0.16, 0.21 ± 0.16, and 0.20 ± 0.16, respectively, with an overall mean of 0.20 across populations. For all genotyped animals, the mean MAF ranged from 0.21 for OAR 11, 12, 14, 2, and 24 to 0.23 for OAR 23. These were lower than the reported average value (0.255 ± 0.136) for New Zealand sheep breeds based on an analysis of 517,902 SNPs and those reported for Corriedale and Merino sheep (0.27), but were higher than the value observed in Creole sheep (Grasso et al., 2014).

**FIGURE 1:**
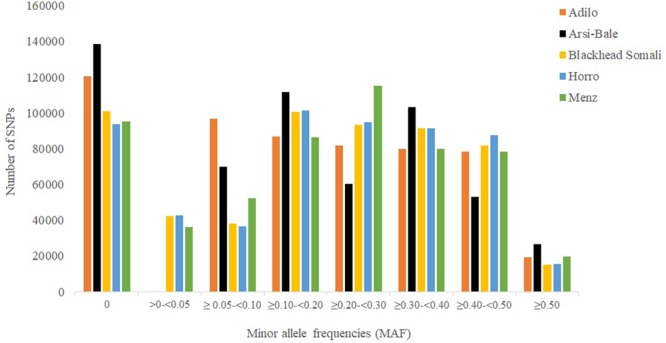
Minor allele distribution across five Ethiopian sheep populations using 563,888 single nucleotide polymorphism (SNPs).

Minor allele frequency distribution for different categories is shown in **Figure 1**. The percentage of fixed SNPs (MAF = 0.00) varied from 16.60% in Horro to 24.60% in Arsi-Bale sheep, with an overall mean of 8.10% across populations, which is lower than that reported for Creole (27%) but higher than those in Merino (3%) and Corriedale (4%) breeds (Grasso et al., 2014). In total, 45,723 fixed SNPs were shared by the five Ethiopian sheep populations; the common SNPs (≥0.10 and ≤0.5) accounted for 71.03% of the total and ranged from 58.03% in Adilo to 66.56% in Horro sheep. On average, highly polymorphic SNPs (MAF ≥ 0.30) accounted for 32.69% of total SNPs and ranged from 31.54% in Adilo to 33.40% in Blackhead Somali sheep. The levels of polymorphic SNPs (80.52%, MAF > 0.01) observed in Ethiopian sheep populations were lower than those observed in Merino (89.4%) and Corriedale (86%) sheep, but were higher than the 69% reported in Creole sheep based on a 50K chip analysis (Grasso et al., 2014). The observed difference between the current and previous studies may be explained by a difference in genotyping platforms and ascertainment bias.

The number of breed-specific SNPs detected for comparison of each breed is given in **Supplementary Table S1**. The highest number of breed-specific SNPs (68,265) was detected in the Menz sheep with frequency ranging from 0.04 to 0.50 and mean of 0.15. The lowest number of breed-specific SNPs (14870) was observed in the Arsi-Bale sheep with frequency ranging from 0.062 to 0.50 and mean of 0.09. Breed-specific SNPs have been detected and used for breed assignment and product traceability in several livestock animals including pigs (Ramos et al., 2011), cattle (Negrini et al., 2009; Ripoli et al., 2013), and sheep (Grasso et al., 2014; Heaton et al., 2014). The population-specific SNPs identified in our Ethiopian sheep populations could be used in a similar manner once they have been validated.

On average, 23,649 (4.19%) loci in the five Ethiopian sheep populations deviated significantly from HWE (*P*< 0.05), with the largest number observed in Adilo (26,348) followed by Blackhead Somali (26,056) sheep. Deviation from HWE is due to inbreeding or genetic substructures within populations (i.e., the Wahlund effect) (Robertson and Hill, 1984; Hart and Clark, 1997; Choi et al., 2009).

The PI_HAT estimated based on 497,294 loci between pairs of individuals was 0.09, 0.03, 0.09, 0.08, and 0.09% for Arsi-Bale, Horro, Adilo, Menz, and Blackhead Somali sheep, respectively, and 0.01% across populations (**Table 2**). HO over all loci (497,292 SNPs) varied from 0.30 in Arsi-Bale, Horro, and Adilo sheep to 0.33 in Menz. The average gene diversity or HE across the five populations was 0.30 and ranged from 0.29 (Arsi-Bale) to 0.32 (Menz). In all populations, HO was higher than or equal to HE, except in Horro sheep. The levels of within-breed genetic variation for Ethiopian sheep populations were within the range reported for New Zealand sheep breeds (0.249–0.383) analyzed using a 600K SNP chip (Brito et al., 2017).

Sample size and the population in which SNPs are detected affect population parameter estimates (Lachance and Tishkoff, 2013; McTavish and Hillis, 2015). Variability is often overestimated in individuals from which the genotyping panel is developed (Rosenblum and Novembre, 2007). We also investigated the effect of ascertainment bias on genetic diversity parameters using loci pruned based on LD. The HE of the unpruned dataset (0.33) was reduced (0.26) after pruning SNPs with high LD within each breed (**Supplementary Table S2**). Removing SNPs in high LD minimizes the effects of ascertainment bias and reduces heterozygosity (Kijas et al., 2012; Edea et al., 2015). In both datasets, estimated inbreeding coefficients (*F*) were negative in all populations, except in Horro sheep (*F* = 0.00–0.02). Overall inbreeding in all populations was estimated as 0.06. The most inbred individual was an Adilo sheep (*F* = 0.30), whereas the most outbred individual was a Blackhead Somali sheep (*F* = –0.33).

### Population Divergence and Relationships

Analysis of molecular variance based on 497,294 autosomal SNPs revealed variations of 5.38% (*P*< 0.0001) and 94.62% among and within populations, respectively. The large within-population variation observed in Ethiopian indigenous sheep populations can be exploited through appropriate breeding strategies to improve productivity. When an analysis was performed for sheep populations grouped based on tail phenotype (long fat-tailed, short fat-tailed, and fat-rumped), among-groups variance was 3.33, with 93.82% within individuals (**Table 3**). Further analysis of populations grouped according to ecological distributions (high- vs. lowland) revealed that 1.28% of the variance was among groups, 4.70% (*P*< 0.0001) among populations within groups, 1.61% among individuals within populations, and 92.47% within populations.

**Table 3:** Analysis of molecular variance of Ethiopian sheep populations grouped based on tail phenotype from analysis of 497,294 SNPs analysis.

Source of variation	Degree of freedom	Sum of squares	Variance components	Percentage of variation
Among groups	2	452560.919	2614.34624	3.33
Among populations within groups	2	244943.771	2229.52922	2.84
Within populations	117	8605746.777	73553.39126	93.82
Total	121	9303251.467	78397.26672	100

When we previously grouped Ethiopian cattle populations based on their ecological distribution (high- vs. lowland), the estimated among-group variation was 0.42% (Edea et al., 2013), which is lower than the value observed here. The variability among Ethiopian sheep populations was higher than the value of 3.64% reported among five Moroccan sheep breeds based on microsatellite markers (Gaouar et al., 2016).

*F*_ST_ values and Reynolds’ genetic distances among the five Ethiopian sheep populations were estimated using 497,294 SNPs (**Table 4**). The overall *F*_ST_ value among the five populations was low (0.053) but significant (*P*< 0.0001). *F*_ST_ for all pairs of populations also differed significantly from zero (*P*< 0.001) and ranged from 0.02 to 0.07, with the closest pairwise value (0.02) observed between Arsi-Bale and Horro sheep. Menz sheep were more distantly related to other Ethiopian sheep populations (*F*_ST_ = 0.05–0.07).

**Table 4:** Pairwise genetic differentiation (*F*_ST_) (below diagonal) and Reynolds’ genetic distance (above diagonal) among five Ethiopian sheep populations based on an analysis of 497,294 SNPs.

Population	Arsi-Bale	Horro	Adilo	Menz	Blackhead Somali
Adilo	0.046	0.029		0.075	0.062
Arsi-Bale		0.017	0.047	0.076	0.066
Blackhead Somali	0.064	0.052	0.060	0.070	
Horro	0.017		0.030	0.053	0.054
Menz	0.073	0.051	0.073		0.073

The average *F*_ST_ among Ethiopian sheep populations was higher than the values reported for Ethiopian cattle (0.01) and goats (0.0245) (Edea et al., 2013; Mekuriaw, 2016), but similar to the mean value of 0.046 obtained using microsatellite markers (Gizaw et al., 2007) and higher than the values in Moroccan [3.6%; (Gaouar et al., 2016)], Algerian [3.8%; (Gaouar et al., 2015)], and Tunisian [3%; (Sassi-Zaidy et al., 2014)] sheep breeds.

### Population Structure

**FIGURE 2:**
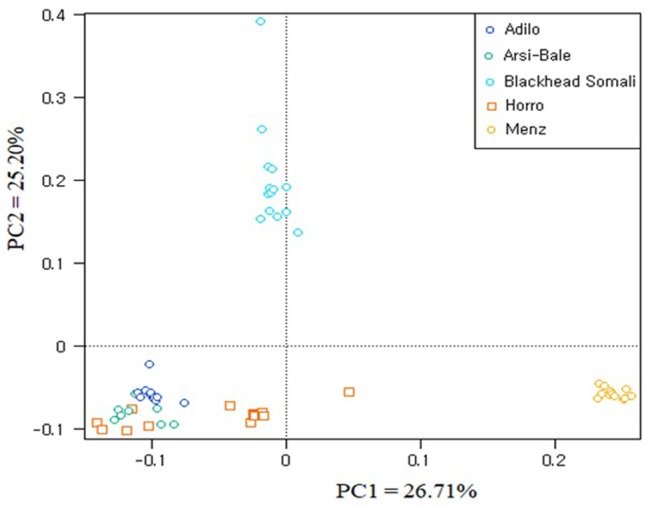
Clustering of individual animals in five Ethiopian sheep populations based on an analysis of 497,294 SNPs.

**FIGURE 3:**
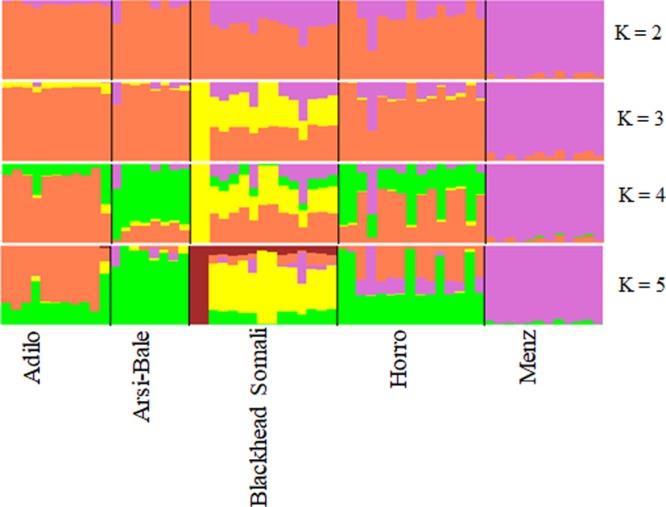
Bayesian-based clustering of five Ethiopian sheep populations based on an analysis of 80,602 SNPs.

To illustrate relationships within individuals and among Ethiopian sheep populations, PCA was performed using 497,294 SNPs. PC1 and PC2 accounted for 26.71 and 25.20%, of the variation, respectively, and clustered the five sheep populations according to their tail phenotypes: long fat-tailed (Arsi-Bale, Horro, and Adilo), short fat-tailed (Menz), and fat-rumped (Blackhead Somali). These clustering patterns corresponded with their geographic distribution. PC1 segregated long-fat-tailed and fat-rumped populations from the short fat-tailed Menz sheep, whereas PC2 separated lowland fat-rumped Blackhead Somali sheep from highland fat-tailed populations (**Figure 2**). Menz sheep formed a tight cluster, whereas outliers were detected in the other populations. The unique genetic background of Menz sheep was corroborated by the STRUCTURE analysis results. At *K* = 2, the three-long fat-tailed sheep populations formed a single group while Menz sheep formed an independent cluster with some admixture from the other populations. Blackhead Somali sheep shared the genetic background of the long fat-tailed populations (**Figure 3**). At *K* = 3, Blackhead Somali sheep tended to segregate, yet shared about 35% of its genome with long fat-tailed populations. The PCA and STRUCTURE analysis revealed clear signatures of admixture among Ethiopian sheep populations—particularly among long-fat tailed breeds—as well as genetic introgression from short-fat tailed Menz into other populations.

Grouping of populations according to tail-phenotype and ecology is in line with the previous microsatellite based analysis (Gizaw et al., 2008). Morphological variation analysis also grouped Ethiopian sheep populations according to their tail-phenotype (long fat-tailed, short fat-tailed and fat-rumped) and ecological distribution [sub-alpine, wet highland and arid lowlands (Gizaw et al., 2008)]. These results further support the independent introduction of fat-tailed and fat-rumped sheep into Africa. Accordingly, it was thought that fat-tailed sheep were introduced into Africa during the third wave of migration following thin-tailed hair sheep and thin-tailed wool sheep, whereas fat-rumped sheep entered much later (Epstein, 1971; Ryder, 1984).

As indicated by our genetic distance, PCA and STRUCTURE results, the Menz sheep showed greater genetic differentiation and clearly separated from the rest of the populations. Differences in allele frequencies between Menz sheep and other populations might have been due to selection for ecological adaptation, differences in migration histories and geographical isolation. Menz sheep are evolved in the cool sub-alpine climate of highlands (2500–3000 m a.s.l.) and are kept for meat and coarse wool production (Wilson, 1991; Tibbo, 2006), and are one of the most primitive coarse-wool breeds imported from Arabia via the Bab-el-Mandeb Strait (Wilson, 1991). It is thought that fat-tailed coarse-wooled sheep were introduced to Africa after thin-tailed breeds about 3,000 years ago (Wilson, 2011) for which adequate time has elapsed for adaptive evolution to take place. Furthermore, historical data show that the Amhara ethnic group of Ethiopia have inhabited altitudes more 2500 m for at least 5 ky (Alkorta-Aranburu et al., 2012). The Menz sheep have migrated to new areas and co-exist with humans for centuries under such extreme environments. On the other hand, fat-rumped Blackhead Somali sheep are well adapted to semi-arid to extremely arid lowlands with high temperatures and sparse and erratic precipitation (Wilson, 2011). The breed is kept for meat production and selected for higher fat deposition on the rump as a source of energy-dense food during prolonged dry spells (Muigai and Hanotte, 2013).

The low genetic differentiation between the two-long fat-tailed populations (Arsi-Bale and Horro) was further supported by our population STRUCTURE analysis results. Arsi-Bale and Horro sheep populations are predominantly maintained by the Ethiopian Oromo ethnic group. In addition to geographic isolation, ethnic, cultural, and religious differences may act as barriers to gene flow that shape population genetic structure (Madrigal et al., 2001). The chances of animal exchange are greater within the same ethnic group or tribe than between any two different ethnic groups or tribes (Gizaw et al., 2007). Arsi-Bale and Horro sheep both inhabit highland environments and face common selective pressures, which may have shaped their genomes in a similar manner. We previously reported that Arsi and Horro cattle had the lowest level of genetic differentiation among examined breeds (Edea et al., 2013); our current results imply that sheep dispersal accompanied that of cattle.

### Genetic Diversity of Ethiopian Sheep Populations and Their Relationships to Global Sheep Breeds

#### Genetic Diversity and Relationships

To compare genetic diversity and trace historical patterns of Ethiopian sheep population structure on a broader geographic scale, we analyzed 41,752 SNPs that overlap between Ovine50SNP and 600K chips. Polymorphic (MAF > 0.01) and highly polymorphic (MAF > 0.30) SNPs accounted for 92 and 37% of SNPs in Ethiopian sheep populations, respectively. These values were lower than those observed for Australian Merino (96 and 45%, respectively), but higher than those for Dorset Horn (89 and 34%, respectively). Using the OvineSNP50 chip, highly polymorphic (MAF > 0.30) SNPs accounted for 50% of the total in Merino and Corriedale sheep and for 36% of the total in Creole sheep (Grasso et al., 2014). The relatively high levels of genomic variability observed in Merino sheep may be partly ascribed to ascertainment bias, as these breeds were used in SNP discovery of the OvineSNP50 chip (Kijas et al., 2012). Despite their small sample size, Ethiopian sheep populations show moderate genetic variability relative to southern African Namaqua, Indian Garole, and Dorset Horn (**Table 2**). However, Ethiopian sheep populations show slightly lower levels of genetic diversity than the presumed ancestral breeds of the Near East (Afshari; HE = 0.34) and northern Africa (HE = 0.35–0.37). Breeds from or close to domestication centers are expected to retain higher allelic diversity than those that migrated farther away (Canon et al., 2006; Peter et al., 2007). The higher diversity estimates in North African as compared to East African breeds can be further explained by the fact that these populations reflect a high degree of admixture between fat- and thin-tailed sheep, as demonstrated by our STRUCTURE analysis. Given its close proximity to the Near East and Mediterranean sea, North Africa served as a gateway for early livestock introduction to the African continent and is considered as a secondary hotspot of genetic variation (Gautier, 2002).

**FIGURE 4:**
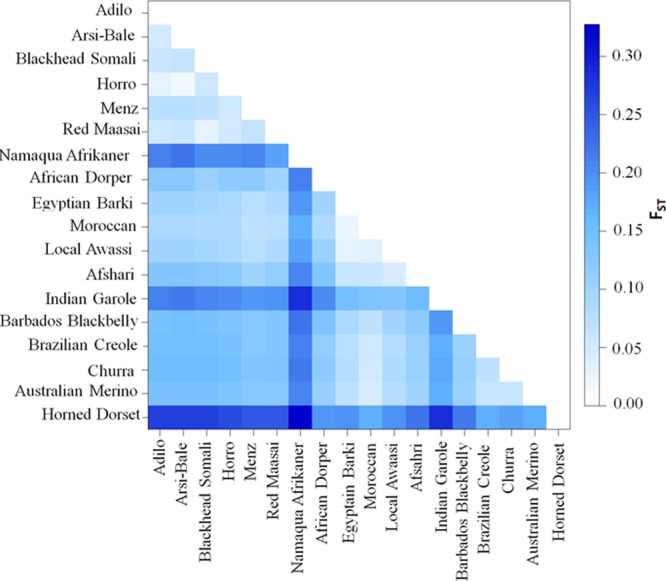
Genetic differentiation (*F*_ST_) values among 18 sheep populations/breeds based on an analysis of 40,770 SNPs.

Pairwise *F*_ST_ (**Figure 4** and **Supplementary Table S3**) and Reynolds’ genetic distances (**Supplementary Table S4**) were calculated between the 18 sheep breeds/populations. The lowest differentiation was in Ethiopian populations (Arsi-Bale and Horro; *F*_ST_ = 0.02) and in North African breeds (Egyptian Barki and Moroccan sheep; *F*_ST_ = 0.02). Pairwise genetic differentiation comparisons revealed that the highest *F*_ST_ value (*F*_ST_ = 0.33) was obtained between the Dorset Horn and Namaqua Afrikaner. Within African sheep breeds, the highest differentiation (mean of 0.21) was observed between Ethiopian and Namaqua Afrikaner breeds. The low within-breed genetic diversity in Namaqua Afrikaner and high genetic differentiation between this breed and other East African sheep populations was likely due to genetic drift, which is consistent with the significantly smaller population size of Namaqua Afrikaner (Qwabe et al., 2013). Ethiopian and North African sheep breeds showed moderate genetic differentiation (*F*_ST_ = 0.08–0.09), while a higher value detected between East African and Middle Eastern breeds (*F*_ST_ = 0.12). It is well documented that the Nile River Valley served as a genetic corridor for human and livestock gene flow between the northern and southern parts of the continent across sub-Saharan Africa (Krings et al., 1999; Horsburgh et al., 2013).

Analysis of molecular variance for the 18 global populations grouped based according to geographical regions (Africa, Asia and western) revealed that 3.68% (*P*< 0.0001) of the variance was among groups, 10.64% among populations within groupings, and 85.69% within populations. The *F*_ST_ value was 0.1431 (*P*< 0.0001), which showed that 14.31% of the total genetic variation was due to population differences. The variation observed among the geographic regions in this study was lower than the reported value of 5.8% (Kijas et al., 2012). To assess genetic differences among the geographic regions within the African continent, we further ran AMOVA by grouping African sheep breeds according to their geographic distribution (North, East, and South). Results indicated that 8.23% (*P* = 0.01) of the variation was among groups and 4.20% among populations within groups. The *F*_ST_ value was 0.1243, which revealed 12.43% of the total genetic difference was attributed to population differences, and the remaining 87.57% accounted by variation within populations.

#### Phylogenetic Cluster Analysis

**FIGURE 5:**
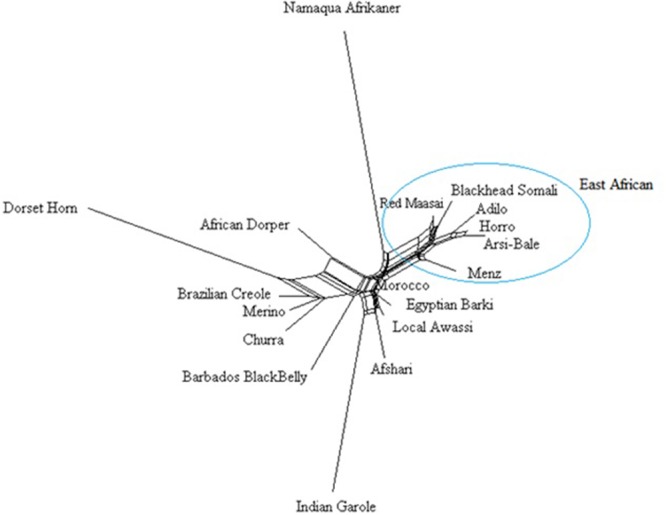
Neighbor network constructed from Reynolds’ genetic distances based on an analysis of 40,770 SNPs.

A Neighbor-Net network constructed using 40, 770 SNPs clustered the study population according to their geographic region (**Figure 5**), with close clustering of breeds or populations within a region. Among Ethiopian sheep, the two highland and fat-tailed sheep (Arsi-Bale and Horro) were closely clustered. Despite the observed phenotypic differences, fat-rumped Blackhead Somali sheep were more closely associated with fat-tailed Red Maasai sheep than with fat-tailed Ethiopian sheep populations. These populations are reared under mobile pastoral and agro-pastoral systems, and there is a high chance of inter-population mating in Kenya (Wilson, 1991). The African Dorper—a composite breed developed from Dorset Horn and Blackhead Persia (Kovács et al., 2008)—was closer to Dorset Horn than to Blackhead Somali, which is a strain of Blackhead Persian sheep.

The Middle Eastern breeds (Afsahri and Awassi) formed another group with Egyptian Barki sheep. In the phylogenetic tree, the Moroccan sheep was positioned in an intermediate position. The Brazilian Creole clustered with the Iberian populations (**Figure 5**). Long branches were noted for Namaqua Afrikaner, Dorset Horn and Indian Garole, possibly due to small effective population size, which concurs with previous reports (Kijas et al., 2009; Spangler et al., 2017). These results are supported by population structure and admixture analyses. Despite the observed significant effect of ascertainment bias on genetic diversity, we did not detect any differences in the phylogenetic tree results for 40,770 and 6163 loci subjected to LD pruning (data not shown). In agreement with our results, it has been demonstrated that increasing the number of loci does not improve the reliability of the phylogenetic tree (Litt and Luty, 1989).

#### Population Structure Analyses

**FIGURE 6:**
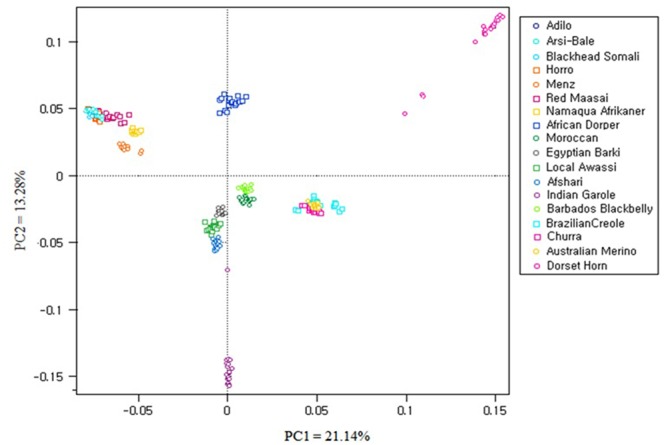
Principal component analysis (PCA) results of 18 sheep breeds from a dataset of 40,770 SNPs.

Principal component analysis was carried out using 40,770 SNPs overlapping between OvineSNP50 and Ovine HD SNPs and the 6163 SNPs left after LD pruning (**Figure 6** and **Supplementary Figure S1**). PC1 accounted 21.14% of the total variation and separated the African breeds, except Moroccan sheep from the Western breeds. Menz and Namaqua Afrikaner were closer to the rest of the East African population but remained as a separate cluster. Eastern and Southern African breeds were separated from the Middle Eastern and North African breeds by PC2. Admixed populations should fall between their two ancestral populations, and the proportion of ancestry inherited from each can be linearly estimated (McVean, 2009). Accordingly, the African composite Dorper was positioned between Dorset Horn and East African populations, while Egyptian Barki sheep were proximal to the Middle Eastern Awassi breed. These results were consistent for 40,770 SNPs and the 6163 SNPs remaining after pruning based on LD, revealing a lack of strong ascertainment bias (**Supplementary Figure S1**).

**FIGURE 7:**
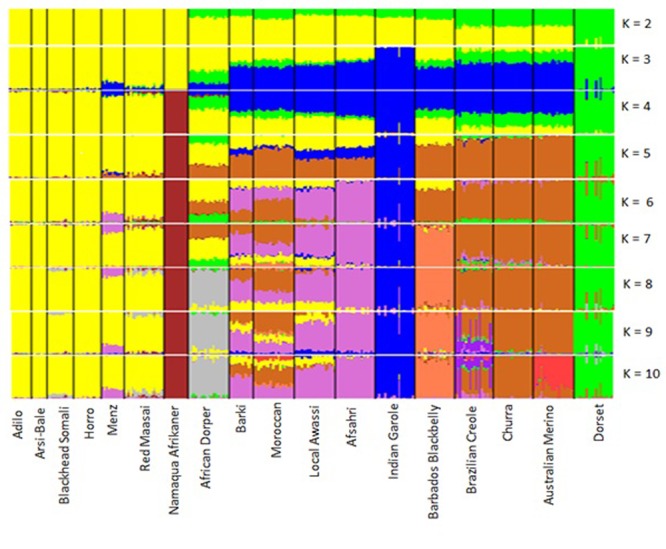
Clustering of 18 sheep breeds based on an analysis of 6163 SNPs for *K* = 2–10.

The results of the structural analysis for hypothetical populations ranging from 2 to 10 are shown in **Figure 7**. At *K* = 2 and *K* = 3, Eastern and Southern African sheep formed one group and Dorset Horn was an independent cluster, which was supported by the PCA results. At *K* = 3, thin-tailed Indian Garole was separated from the other breeds. From *K* = 4–10, Namaqua Afrikaner sheep clearly segregated from East African populations, which was well supported by the phylogenetic results. Northern Africa is mostly populated by fat-tailed sheep (Muigai and Hanotte, 2013), but our STRUCTURE analysis revealed substantial signatures of admixture in the genomes of North Africa populations as compared to their Eastern and Southern African counterparts. This is in accordance with the historical introduction of sheep into Africa and their dispersion across the continent through the Nile Valley; for instance, thin-tailed sheep spread into the Western Sahara via northern Africa (Muigai and Hanotte, 2013), which may have left its genomic legacy in today’s North African sheep populations.

The low genetic background of Asiatic and Iberian thin-tailed sheep detected in fat-tailed East and South African breeds is consistent with the distinct histories and non-overlapping geographic distributions of these populations (Muigai, 2003b), and support the predominance of fat-tailed sheep in the eastern and southern parts of Africa (Muigai and Hanotte, 2013). Archeological evidence traces the first fat-tailed sheep to the Eastern Ethiopian highlands (Clark and Williams, 1978). Moreover, analyses of autosomal markers and the Y chromosome have revealed the distinct evolutionary histories of thin- and fat-tailed African sheep breeds (Muigai, 2003a; Aswani, 2007).

At *K* = 8, we observed a divergence of the African Dorper from the East African populations, which was also well supported by our PCA and Neighbor-Net network results. At *K* = 6–10, Menz sheep shared 20–22% its genome with Middle Eastern fat-tailed sheep, whereas this value did not exceed 1% in the remaining Ethiopian sheep populations. The influence of Middle Eastern fat-tailed sheep detected in Menz can be explained by the fact that within Menz and adjacent areas, cross-breeding between Menz and Awassi populations has been ongoing for more than three decades (Gizaw and Getachew, 2009). At the optimum *K*-value of 10, Red Maasai shared between 8 and 10% of its genomes with African Dorper. It is well known that the Dorper breed was introduced into Kenya in the 1960s and was indiscriminately crossed with local breeds including Red Maasai to increase meat production in local sheep populations (Verbeek et al., 2007). Similarly, Blackhead Somali—which is a strain of Blackhead Persian sheep—was used as a maternal line in the development of African Dorper (Wilson, 1991). The sizeable genetic admixture between Iberian and North African breeds, particularly with Moroccan sheep was clearly illustrated at *K* = 5–9. This finding mirrors historical human and livestock movements between Northern Africa and the Iberian Peninsula (Boone and Benco, 1999; Botigué et al., 2013); archeological and DNA evidence demonstrates the influence of North African domestic livestock species on indigenous populations of the Iberian Peninsula (Beja-Pereira et al., 2002; Anderung et al., 2005).

The close clustering of East African sheep populations and distinct separation from their northern counterparts was well demonstrated by our phylogenetic, PCA, and STRUCTURE analyses. This result coincides with the evidence that fat-tailed sheep were introduced into Africa via two independent routes: the Horn of Africa and northern Africa from the Middle East (Ryder, 1984). The lowest genetic differentiation obtained for the two Ethiopian sheep populations (Arsi-Bale and Horro; *F*_ST_ = 0.02) was also well supported by population STRUCTURE and Neighbor network analyses. We suggest that this could be due to gene flow and similarity of production environments (Gizaw et al., 2007). On the other hand, the unique genetic composition of short fat-tailed Menz sheep is consistent with its distinct phenotypes, population histories, and ecological distribution (Gizaw et al., 2007).

## Conclusion

Our high-density genome-wide SNP analyses revealed that Ethiopian sheep populations are roughly clustered according to their geographic distribution and tail phenotype. The genetic diversity and structure of Ethiopian sheep populations can be explained by historical events and selection for ecological adaptation. The high-density SNP data generated in this study can be used to identify genes and pathways relevant for physiological adaptation to extreme environments and variation in phenotypic traits. The close clustering of Eastern African breeds and their separation from North African breeds provide evidence that fat-tailed sheep were introduced to the continent via the Horn of Africa and migrated further southwards. Additional genome-wide analyses of thin-tailed sheep breeds from Eastern and Western Africa and fat-tailed breeds from the Arabian Peninsula can clarify the evolutionary history of sheep on the African continent and provide new insight into the genomic landscape of African sheep breeds.

## Data Accessibility

Genotypic data of 72 animals representing five Ethiopian sheep populations are deposited and available at (https://www.animalgenome.org/repository/pub/KORE2017.1122/).

## Ethics Statement

Local regulations were observed. This research used Nasal swab DNA collection kits, which does not require injure the animal nor impose pain.

## Author Contributions

ZE and K-SK conceived the study, analyzed the data, and wrote the manuscript. TD provided logistical support for field data collection and facilitated sample export. HD and K-TD revised the manuscript. All authors read and approved the final manuscript.

## Conflict of Interest Statement

The authors declare that the research was conducted in the absence of any commercial or financial relationships that could be construed as a potential conflict of interest.

## Footnotes

1 http://www.sheephapmap.org/download.php

2 http://projects.ensembl.org/nextgen/

3 www.goldenhelix.com

## Supplementary Material

FIGURE S1 Results of PC 1 and 2 from the dataset of 6163 SNP markers.

TABLE S1 The number of private alleles detected in the comparison of each sheep population.

TABLE S2 Diversity indices in 5 Ethiopian sheep populations estimated from 80,602 SNPs obtained after LD pruning.

TABLE S3 Genetic differentiation (FST) between the study sheep breeds based on analysis of 40,770 SNPs.

TABLE S4 Reynolds’ genetic distance between the 18 sheep breeds based on analysis of 40,770 SNPs.
