# Supplementary figures and images for: Genetic Diversity and Population Structure of Ethiopian Sheep Populations Revealed by High-Density SNP Markers
Source: Front Genet. 2017 Dec 22;8:218. doi: 10.3389/fgene.2017.00218 (PMC5744078; doi:10.3389/fgene.2017.00218)

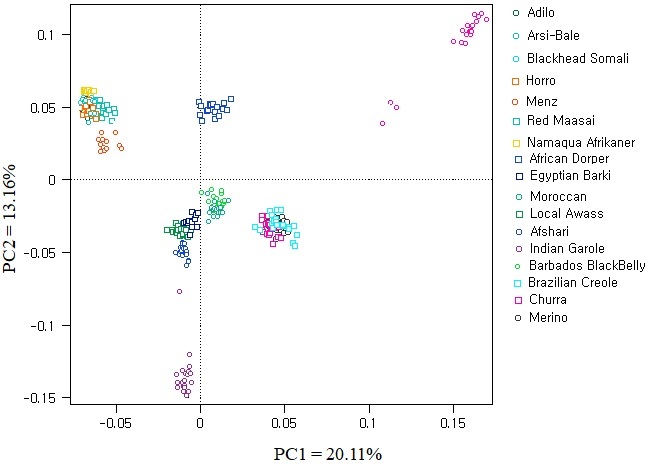

Supplement: FIGURE S1 — Results of PC 1 and 2 from the dataset of 6163 SNP markers. [file Image_1.jpg]
