# Supplementary material for: Genetic Diversity and Population Structure of Ethiopian Sheep Populations Revealed by High-Density SNP Markers
Source: Front Genet. 2017 Dec 22;8:218. doi: 10.3389/fgene.2017.00218 (PMC5744078; doi:10.3389/fgene.2017.00218)
Supplement: TABLE S1 — The number of private alleles detected in the comparison of each sheep population. [file Table_1.DOCX]

Table S1. The number of private alleles detected in the comparison of each sheep population

| Population pair | Specific to | Number of SNP | Allele frequency |  |  |
| --- | --- | --- | --- | --- | --- |
|  |  |  | Mean | Minimum | Maximum |
| Adilo-Arsi | Adilo | 49942 | 0.13 | 0.045 | 0.50 |
| Adilo-BHS | Adilo | 27566 | 0.12 | 0.045 | 0.50 |
| Adilo-Horro | Adilo | 19134 | 0.10 | 0.045 | 0.50 |
| Adilo-Menz | Adilo | 28854 | 0.13 | 0.045 | 0.50 |
| Arsi-Adilo | Arsi | 31663 | 0.12 | 0.062 | 0.50 |
| Arsi-BHS | Arsi | 24688 | 0.14 | 0.062 | 0.50 |
| Arsi-Horro | Arsi | 14870 | 0.09 | 0.062 | 0.50 |
| Arsi-Menz | Arsi | 24785 | 0.15 | 0.062 | 0.50 |
| BHS-Adilo | BHS | 46972 | 0.11 | 0.033 | 0.50 |
| BHS-Arsi | BHS | 62374 | 0.12 | 0.030 | 0.50 |
| BHS-Horro | BHS | 29676 | 0.10 | 0.033 | 0.50 |
| BHS-Menz | BHS | 33861 | 0.11 | 0.033 | 0.50 |
| Horro-Adilo | Horro | 45855 | 0.09 | 0.033 | 0.50 |
| Horro-Arsi | Horro | 59870 | 0.10 | 0.033 | 0.50 |
| Horro-BHS | Horro | 36991 | 0.10 | 0.033 | 0.50 |
| Horro-Menz | Horro | 33305 | 0.10 | 0.033 | 0.50 |
| Menz-Adilo | Menz | 54055 | 0.14 | 0.04 | 0.50 |
| Menz-Arsi | Menz | 68265 | 0.15 | 0.04 | 0.50 |
| Menz-BHS | Menz | 39656 | 0.13 | 0.041 | 0.50 |
| Menz-Horro | Menz | 31785 | 0.11 | 0.04 | 0.50 |

BHS = Blackhead Somali
