# Supplementary material for: Genetic Diversity and Population Structure of Ethiopian Sheep Populations Revealed by High-Density SNP Markers
Source: Front Genet. 2017 Dec 22;8:218. doi: 10.3389/fgene.2017.00218 (PMC5744078; doi:10.3389/fgene.2017.00218)
Supplement: TABLE S2 — Diversity indices in 5 Ethiopian sheep populations estimated from 80,602 SNPs obtained after LD pruning. [file Table_2.DOCX]

| **Breed/population** |  | **Parameter** | | |
| --- | --- | --- | --- | --- |
|  | **n** | **HO** | **HE** | **F** |
| Arsi-Bale | 8 | 0.24 | 0.23 | −0.05 |
| Horro | 15 | 0.25 | 0.25 | 0.00 |
| Adilo | 11 | 0.25 | 0.24 | −0.02 |
| Menz | 12 | 0.27 | 0.26 | −0.06 |
| Blackhead Somali | 15 | 0.26 | 0.25 | −0.06 |
| **Overall** | **61** | **0.26** | **0.26** | **0.03** |

**Table S2.** Diversity indices in 5 Ethiopian sheep populations estimated from 80,602 SNPs obtained after LD pruning
